# Minimally Invasive Tubular Posterior Cervical Laminectomy in Medically Complex Patients: A Case Series With Clinical and Radiographic Outcomes

**DOI:** 10.7759/cureus.103622

**Published:** 2026-02-14

**Authors:** Kareem Khalifeh, Timothy Y Kim, Brian Hirshman, Martin Pham

**Affiliations:** 1 Neurological Surgery, University of California, San Diego School of Medicine, San Diego, USA

**Keywords:** case series, cervical, laminectomy, minimally invasive, myelopathy, tubular

## Abstract

Objectives

Minimally invasive (MIS) techniques have become increasingly favored for cervical decompression. Among them, tubular posterior cervical laminectomy represents a promising option for patients with significant medical comorbidities. However, radiographic outcomes associated with this technique have not been fully described. The objective of this study is to evaluate clinical and radiographic outcomes of this approach in medically complex patients.

Materials and methods

This retrospective case series examined all patients who underwent an MIS tubular cervical laminectomy for bilateral decompression by a single surgeon. Demographic, clinical, and radiographic outcomes were collected.

Results

Eighteen patients (12 (67%) male) were identified with a mean age of 76 years, a mean body mass index of 28 kg/m^2^, and mean follow-up of 23 months (range 3-40). All patients had cervical stenosis with additional diagnoses of cervical myelopathy (n=15 (83%)), adjacent segment disease (n=3 (17%)), syringomyelia (n=1 (6%)), and dorsal epidural abscess (n=1 (6%)). Mean American Society of Anesthesiologists class was 3.2 (range 2-4) with comorbidities including coronary artery disease (n=5 (28%)), congestive heart failure (n=2 (11%)), and aortic stenosis (n=2 (11%)). Median operative levels were 2 (range 1-3). Mean operating skin-to-skin time was 79 minutes (one level), 112 minutes (two levels), and 146 minutes (three levels) with estimated blood loss <50 cc in all cases. Eleven (79%) of 14 elective patients were discharged the same day, with three patients (21%) admitted for rehabilitation disposition planning. Mean improvement in the radiographic anterior-posterior central canal distance was 4.9 mm (p<0.05) and axial cross-sectional area was 71.9 mm^2^ (p<0.05). Modified Japanese Orthopaedic Association scores showed improvements of a mean preoperative 10.8 to mean postoperative 12.3 (p<0.05). There were no perioperative complications or reoperations during the follow-up period.

Conclusions

MIS tubular cervical laminectomy demonstrated successful clinical and radiographic outcomes in this case series. This approach should be considered as a safer alternative to open surgeries, especially in medically complex patients.

## Introduction

Cervical spondylotic myelopathy (CSM) is an increasingly common insidious neurologic condition characterized by degenerative changes of the spine, resulting in compression of the spinal cord and surrounding structures [1]. Although its exact pathophysiology remains unclear, clinical symptoms are often associated with disc herniation, osteophyte formation, and/or posterior cord compression from facet and ligamentum flavum hypertrophy [2]. Cervical myelopathy is most commonly managed surgically, and depending on the patient and pathology profile, various techniques can be employed to decompress the spinal cord [3].

Surgical management of cervical myelopathy is performed to arrest progression of neurological deficits. With the advancement of minimally invasive (MIS) techniques, instrumentation, and technology, tubular “over-the-top” laminectomy has emerged as a promising procedure for cervical decompression in a variety of patient populations [2,4-9]. Initially applied in the treatment of lumbar spinal stenosis, the technique has since been adopted for the management of CSM and other etiologies of cervical stenosis. Despite this adaptation, cervical tubular laminectomy remains less well described than anterior cervical discectomy and fusion (ACDF) or open laminectomy with or without fusion, in part due to its relative novelty and the technical challenges of operating in the cervical spine. Furthermore, comprehensive radiographic outcomes are not fully described in the literature. As such, the aim of this case series is to retrospectively review clinical and radiographic outcomes associated with this approach for cervical decompression in a series of medically complex patients and discuss technical considerations, clinical implications, and limitations.

## Materials and methods

Study design and patient selection

A retrospective case series was conducted of patients who underwent MIS unilateral tubular “over-the-top” cervical laminectomy for bilateral decompression by an attending neurosurgeon at a single academic center between February 2022 and February 2024. Inclusion criteria included patients with symptomatic cervical stenosis and those who were over 18 years old. Patients were excluded if any portion of the operation included open exposure or instrumentation and fusion. Eighteen patients met the criteria for this study. This case series has been reported in line with the PROCESS Guideline [10].

Outcome measures

Data points collected include demographics (age, sex, body mass index (BMI)), operative measures (number of levels, operative time, estimated blood loss (EBL), complications) and pre- and postoperative clinical and radiographic results. These data include American Society of Anesthesiologists (ASA) preoperative physical status, hospital length of stay (LOS), average outpatient follow-up, change in anterior-posterior central canal (AP) distance, change in the spinal cord cross-sectional area (CSA), and change in modified Japanese Orthopaedic Association (mJOA) score [11]. Radiographic measurements were done by the senior author who is a fellowship-trained, board-certified neurosurgeon with a subspecialty in complex and minimally invasive spine surgery. These calculations were conducted using a Picture Archiving and Communication System (PACS) machine capable of multiplanar reconstructions which allowed for true AP and volumetric surface area analysis. The central canal AP distance and spinal cord CSA were measured using the Lee criteria [12].

Statistical methods

A one-tailed paired two-sample t-test was calculated for AP distance, CSA, and mJOA score data to assess whether the differences between pre- and postoperative scores were statistically significant. These calculations were performed using the Microsoft Excel spreadsheet software (version 16.95.4) with a confidence interval (CI) set at 95% (alpha=0.05). Results are reported in the following format: t(degrees of freedom) = t statistic, p=p value.

Operative technique

**Figure 1:**
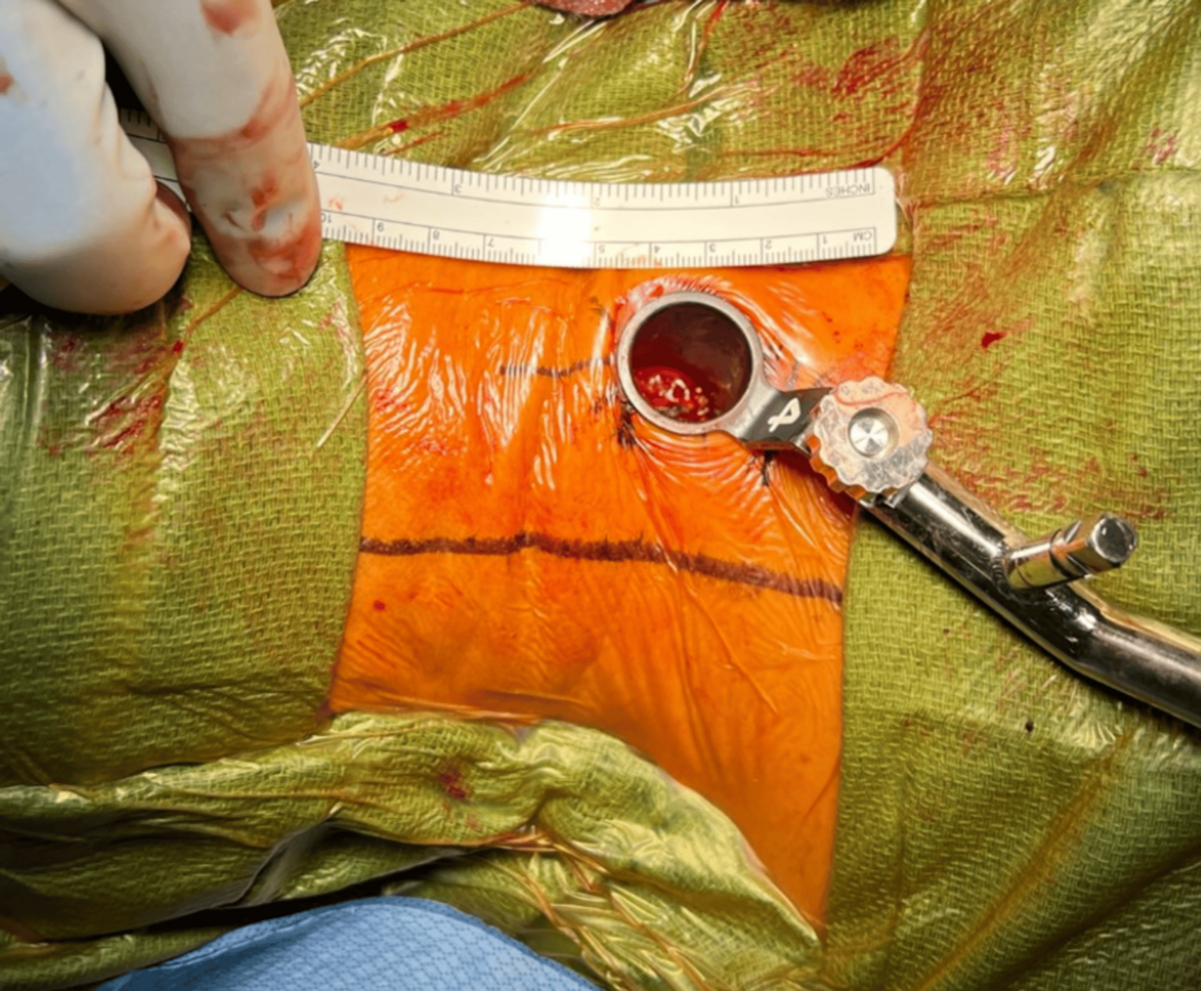
Operative field view of a right-sided minimally invasive cervical laminectomy approach using an 18-mm tubular retractor secured to a table-mounted system.

The operative technique used at our institution has been previously described [13]. The patient is positioned prone on a Jackson table with the head in reverse Trendelenburg. An incision is marked approximately 2.5 cm off midline, and once prepped and draped, this localization is confirmed on fluoroscopy using a spinal needle directed at the more laterally positioned bony facet joint. A 2-cm linear incision is made, and the multiple fascial layers are sharply incised until blunt dissection with the first dilator confirms tactile docking at the lamino-facet junction. While prior techniques have described the use of a K-wire, we have chosen to start only with the first blunt dilator against the bony lamino-facet junction out of concern for entry into the canal or foramen with the thin profile of a K-wire. The first dilator then acts as a tactile "finger" to ensure that a safe bony surface has been identified. Serial dilation is then performed up to a 16- or 18-mm tubular retractor, which is then secured to a table mount system (Figure 1).

Localization at the lower cervical levels can be challenging, especially in larger patients. In these scenarios, we rely on correlating an AP fluoroscopic image with an appropriate Ferguson tilt so that the cervicothoracic junction at C7-T1 is identified, and we count upwards from that junction. We then use lateral fluoroscopy with tight circle collimation to ensure the appropriate trajectory. Another option would be the placement of a navigation array and the use of intraoperative CT for real-time computer-assisted navigation. This would be an excellent adjunct and helpful for surgeons less experienced with this approach.

Monopolar electrocautery is then used to remove any remaining soft tissue and muscle over the bony anatomy. The inferior half of the cranial lamina, superior half of the cauda lamina, lamino-facet junction, and base of the spinous process should all be recognized and identified. Drilling then begins cranially at the ipsilateral lamina until the cranial insertion points of the ligamentum flavum are identified. This drilling is subsequently carried caudally until the caudal insertion point of the ligamentum flavum is identified. With the ligamentum still intact, the tubular retractor is repositioned, aiming more medially, and the contralateral “over-the-top” decompression is performed with removal of the internal ventral lamina above the ligamentum flavum. This is carried out contralaterally both cranially and caudally, following the already known insertion points of the ligamentum flavum.

**Figure 2:**
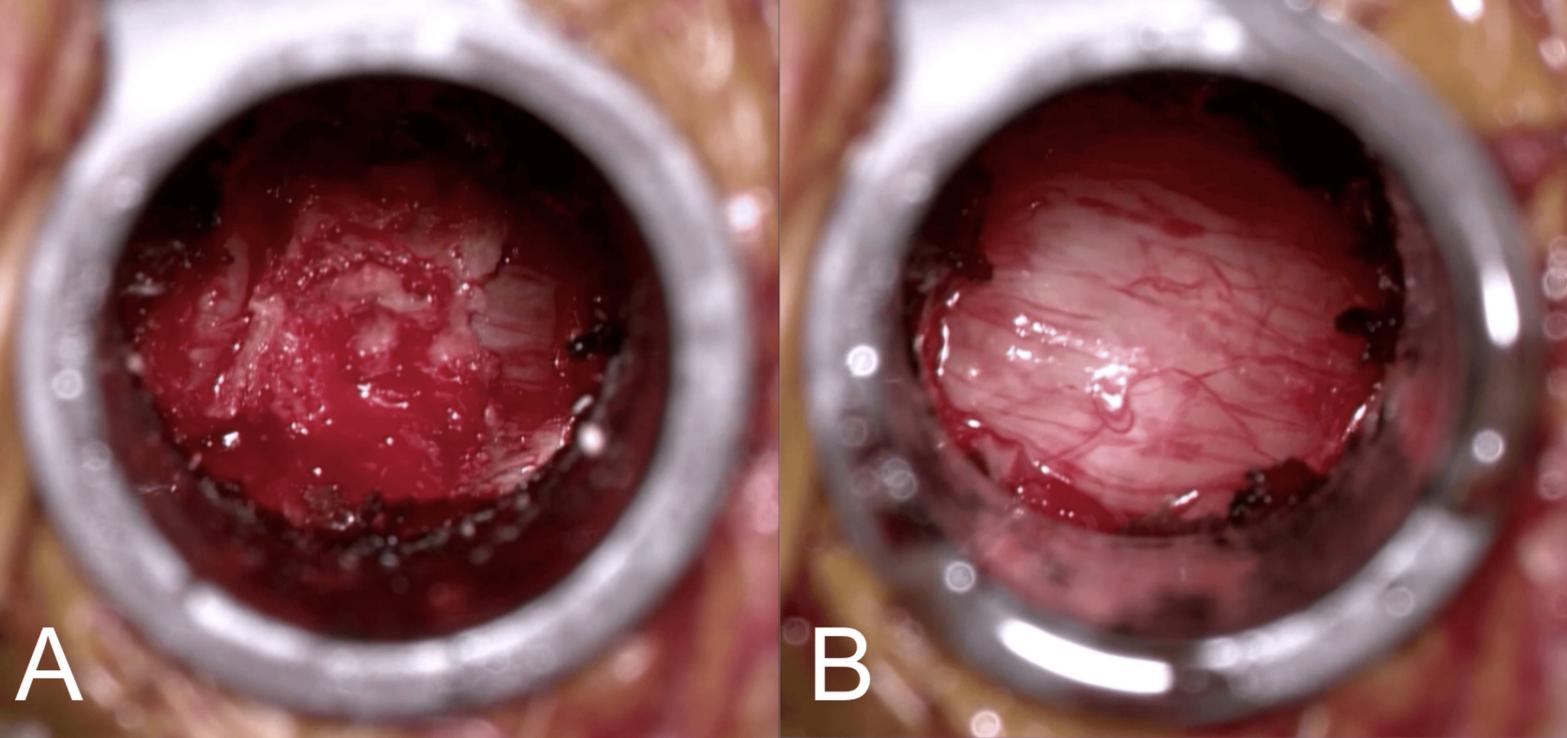
(A) Skeletonized ligamentum flavum showing the exposed cranial and caudal insertion points and the “napkin ring” appearance of the dorsal compression of the cervical dura. (B) After the ligamentum is removed in near-en bloc fashion, the cervical dura is now noted to be well decompressed.

Once the bony decompression is complete, the compression napkin ring band of ligamentum flavum should be appreciated (Figure 2). This is released ipsilaterally with a combination of upcoming curettes and Kerrison punches, and contralaterally with a combination of straight curettes and pituitary rongeurs until the entirety of the ligamentum has been removed and the dura is fully decompressed. Great care is taken to ensure there is never any downward pressure against the cervical dura and spinal cord.

Not unexpected in situations of stenosis, there may be engorged epidural veins that bleed into the field. Hemostasis techniques can be more challenging due to the smaller operative corridor working above the spinal cord and dural sac. Our methods focus on the use of bipolar electrocautery if epidural veins are seen or the use of Surgiflo (Ethicon) with gentle pressure using a small cottonoid.

The wound is then irrigated, hemostasis is achieved, and a multilayer closure is performed. Due to the small incision and ability for controlled hemostasis, we have not found any need to leave a surgical drain, although one can be placed based on the surgeon’s judgment and patient’s morbidities.

## Results

Eighteen patients (12 (67%) male, 6 (33%) female) underwent MIS unilateral tubular cervical laminectomy. The mean age was 76 years (standard deviation (SD): 11.0; range 59-97), the mean BMI was 28 kg/m^2^ (SD: 5.7; range 17.5-40.2), and the mean follow-up was 23 months (range 3-40). Three patients (17%) had only a three-month follow-up and could not be reached for additional contact. Median laminectomy levels performed were 2 (range 1-3). All patients had cervical stenosis with additional diagnoses of cervical myelopathy (n=15 (83%)), adjacent segment disease (n=3 (17%)), syringomyelia (n=1 (6%)), and dorsal epidural abscess (n=1 (6%)). Mean American Society of Anesthesiologists (ASA) class was 3.2 (SD: 0.6; range 2-4) with comorbidities including coronary artery disease (n=5 (28%)), congestive heart failure (n=2 (11%)), and aortic stenosis (n=2 (11%)).

Mean operating skin-to-skin time was 79 minutes for one-level procedures, 112 minutes for two-level procedures, and 146 minutes for three-level procedures with an EBL <50 cc in all cases. Eleven (79%) out of 14 elective patients were discharged the same day as surgery, with three patients (21%) admitted for rehabilitation disposition planning. The remaining four patients presented through the emergency department and continued their hospital stay.

**Figure 3:**
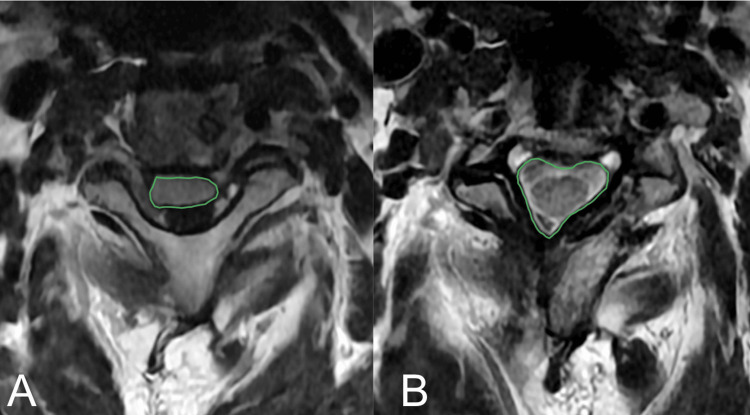
(A) Preoperative and (B) postoperative axial MRI images showing good decompression and reconstitution of spinal canal volume and CSF signal around the entirety of the cervical spinal cord. The green lines denote the spinal cord CSA capturing the dural sac only. CSA: Cross-sectional area

**Figure 4:**
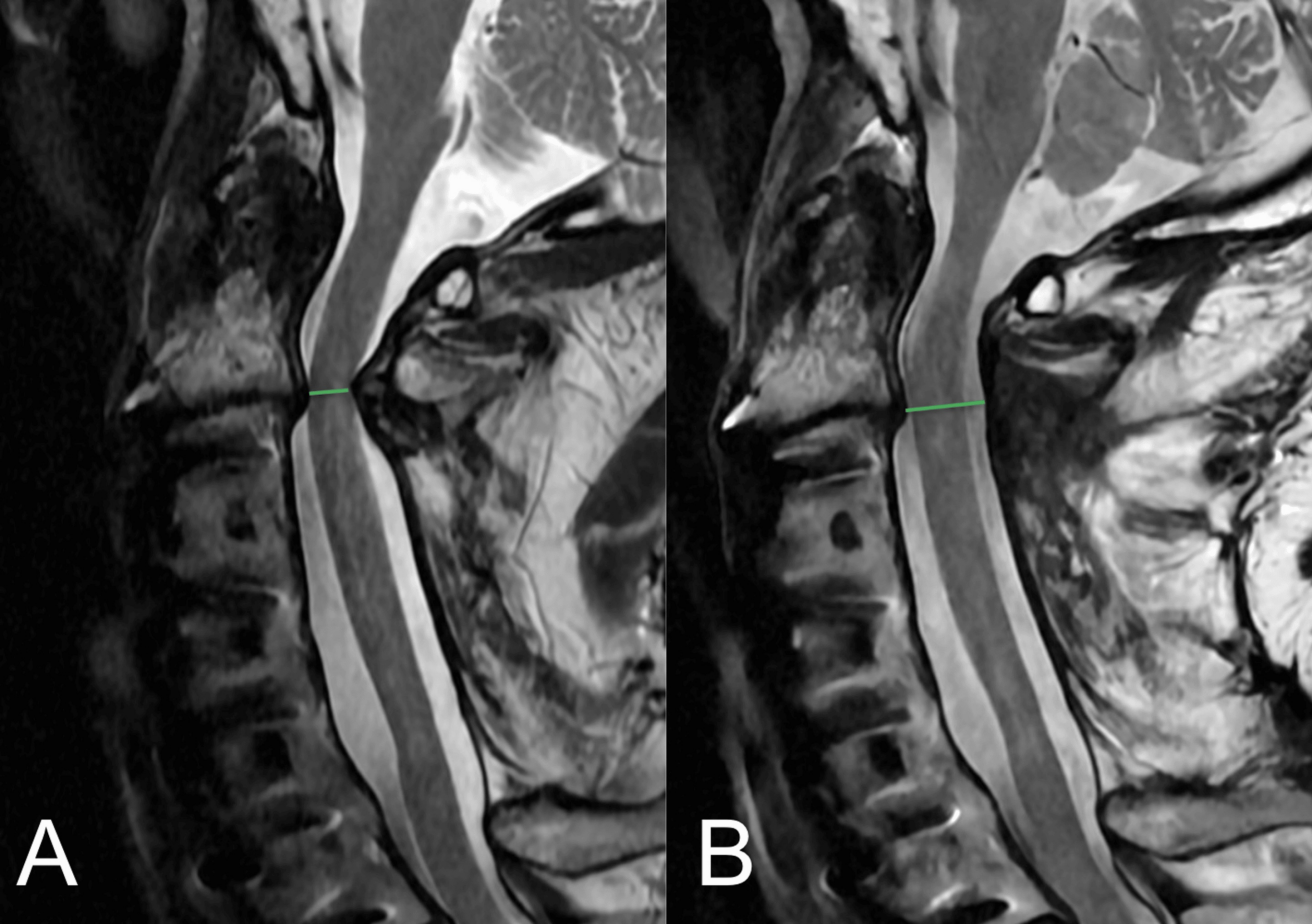
(A) Preoperative and (B) postoperative sagittal MRI images showing good decompression and removal of a primarily dorsally compressive ligamentum flavum. The green lines denote the AP central canal distance measured from the posterior disc in an orthogonal perpendicular line to the dorsal dura.

**Figure 5:**
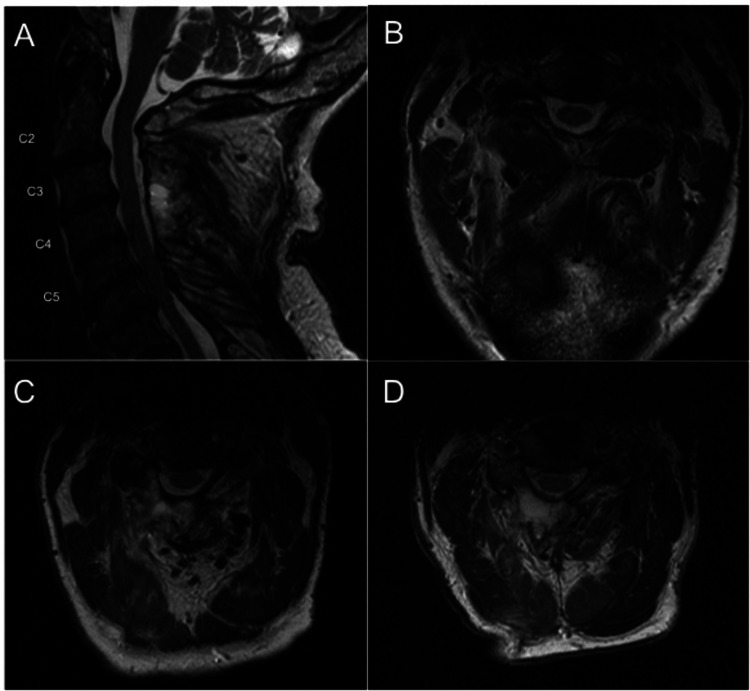
(A) Mid-sagittal T2-weighted MRI of the cervical spine status post three-level decompression with axial views at (B) C2-3, (C) C3-4, and (D) C4-5.

Radiographic data were available for eight patients. Mean improvement in the radiographic AP central canal distance was 4.9 mm (SD: 1.2; t(7)=-11.5, p=4.2x10^-6^ <0.05) and axial CSA was 71.9 mm^2^ (SD: 25.9; t(7)=-7.8; p=5.1x10^-5^ < 0.05) (Figures 3, 4). mJOA scores were available for 12 patients and showed improvement of a mean preoperative 10.8 (range 6-15) to a mean postoperative of 12.3 (range 8-16). The difference between samples was statistically significant with t(11)=-3.4, p=0.003< 0.05. There were no perioperative complications or reoperations during the follow-up period (Table 1). An example of a three-level decompression using this technique can be seen on magnetic resonance imaging (MRI), as shown in Figure 5.

**Table 1:** Clinical and radiographic outcomes. mJOA: Modified Japanese Orthopaedic Association; SD: standard deviation p-value <0.05 was determined to be significant (*)

	Preoperative	Postoperative	Difference	p-value
Mean mJOA score (SD)	10.8 (2.9)	12.3 (2.3)	0.9 (1.3)	0.003*
Mean AP central canal distance (SD) – mm	5.4 (0.9)	10.3 (0.9)	4.9 (1.2)	4.2 × 10^-6^*
Mean axial cross-sectional area (SD) – mm^2^	90 (20.9)	161.9 (39.4)	71.9 (25.9)	5.1 × 10^-5^*

## Discussion

Surgical intervention has classically been the gold standard to improve symptoms and arrest disease progression of cervical myelopathy, and various techniques can be employed including ACDF, anterior cervical corpectomy and fusion, anterior arthroplasty, posterior cervical laminectomy with or without fusion, and posterior cervical laminoplasty. With consideration of patient-specific factors and disease severity, each approach expresses its own risk and advantage profile.

ACDF in particular has become the mainstay form of CSM management due to its ability to decompress the anterior spinal canal and significantly improve neurological outcomes in an MIS manner [14]. However, a database study conducted by Veeravagu et al. reported an overall ACDF complication rate of 15.6% for patients with CSM, and an even higher rate for patients older than 65 years [15]. Open posterior techniques such as laminectomy, laminectomy with fusion, and laminoplasty have also been implemented, especially in multilevel cases. However, these approaches damage the paraspinal muscles and their attachments which impairs the healing process, results in longer hospitalizations, and may result in chronic neck pain [16,17]. Furthermore, open posterior cervical techniques are associated with a range of complications related to instrumentation, C5 palsy, spring-back closure of lamina, and postlaminectomy kyphosis [18]. Complication rates for open posterior laminectomy and fusion have been described as high as 29.2%, and even higher in patients older than 65 years [15].

More recently, MIS posterior techniques have been developed, which preserve the posterior musculature and tension band while also requiring less bony removal than open approaches. Among these, MIS tubular “over-the-top” laminectomy, adopted from its initial application in the lumbar spine, has emerged as a potential management for CSM. Older, sicker, or more medically complex frail patients who may not be good candidates for open cervical exposures or cervical fusions may still greatly benefit from decompression through these lower risk MIS approaches.

In a retrospective case series by Hernandez et al., 15 patients with CSM who were treated with MIS tubular cervical laminectomy experienced significant improvement (p<0.001) in mean visual analogue scale, neck disability index, and mJOA scores. Mean operative time was 125.3 minutes (81.7 minutes per level), and mean EBL was 57.3 cc. No complications were reported [2]. Similarly, Ross & Ross described 30 patients with CSM who underwent MIS tubular cervical laminectomy and experienced significantly improved mJOA scores (p<0.001). Mean operative time was 142 minutes, and EBL was described as minimal. The authors reported no wound infections, durotomies, or readmissions within 90 days of surgery. One patient had an unexplained neurological deficit that resolved over six months [5]. Finally, Ross & O’Glasser reported the use of the same technique in three elderly patients (mean age of 73 years) and also demonstrated successful clinical outcomes with no complications and a mean operative time of 185 minutes. The patients described in this case report highlight MIS tubular cervical laminectomy as a suitable option for elderly and medically fragile patients who would not be otherwise candidates for major surgical procedures [4]. 

Our experience with MIS tubular cervical laminectomy largely reflects the outcomes highlighted in the limited existing body of literature describing this procedure. Our patient population was notably elderly, with a mean age of 76.1 and a maximum age of 97, with a mean ASA class of 3.2, many of whom also endorsed pre-existing cardiac conditions. Nevertheless, they experienced significantly increased mJOA scores (p<0.05), and there were no complications or readmissions in our follow-up period. The patients also exhibited significant improvement in radiographic AP central canal distance and axial CSA (p<0.05), which radiographically confirms spinal cord decompression through this technique. Operative time, EBL, and complication rates were similar to the values reflected in existing studies.

In summary, this technique represents a safe and effective alternative for elderly and medically complex patients with cervical myelopathy who may not tolerate more invasive procedures. Future research should focus on prospective multi-center studies to validate these findings across institutions, establish standardized patient selection criteria, and evaluate long-term biomechanical outcomes and durability of decompression. Comparative effectiveness studies directly comparing MIS tubular approaches to ACDF and open posterior techniques in matched cohorts would further clarify the optimal role of this technique in the surgical management of cervical myelopathy.

Limitations

We acknowledge the relatively small sample size of this study, which limits generalizability. Furthermore, only 44% and 67% of patients received radiographic and clinical follow-up, respectively. Extending the follow-up intervals would provide a clearer understanding of patients' clinical responses and allow for a more thorough evaluation of potential complications and segmental progression of spondylosis. Larger retrospective series and cohort studies comparing the efficacy and safety of this procedure to other traditional forms of management for CSM are certainly warranted and would address these limitations. Likewise, not all patients may be good candidates for this technique, notably patients with primarily anterior compressive etiologies or significant congenital stenosis. However, given the relative infancy of the procedure described and the consistency of our outcomes with that of preexisting literature, we are encouraged that the results outlined in this case series still showcase a role for MIS tubular cervical laminectomy in the described patient demographic.

## Conclusions

Minimally invasive tubular cervical laminectomy demonstrated successful clinical and operative outcomes in this case series consisting of a relatively elderly patient population with significant medical comorbidities. These findings further support this technique as a safe and effective treatment option while highlighting the radiographic outcomes achieved through this approach.
